# Validität des Schweregradindex bei nichtspezifischen chronischen Rückenschmerzen

**DOI:** 10.1007/s00482-024-00844-8

**Published:** 2024-11-07

**Authors:** Petra Hampel, Anna Maria Hüwel

**Affiliations:** Institut für Gesundheits- und Ernährungswissenschaften, Europa-Universität Flensburg, Auf dem Campus 1, 24943 Flensburg, Deutschland

**Keywords:** Stationäre verhaltensmedizinisch-orthopädische Rehabilitation, Schmerzschweregrad, Schmerzspezifische Selbstwirksamkeit, Depressivität, Arbeitsbezogene Risikofaktoren, Inpatient multidisciplinary rehabilitation, Pain grading, Pain self-efficacy, Depressive symptoms, Work-related factors

## Abstract

**Hintergrund:**

Höhere Schmerzgrade hängen mit hoher psychischer Belastung zusammen und erhöhen das Risiko für die Aufrechterhaltung von chronischen Rückenschmerzen (CRS).

**Ziel der Arbeit:**

Die Kriteriumsvalidität des Schweregradindex sollte erstmalig im Kontext der verhaltensmedizinisch-orthopädischen Rehabilitation (VMO) sowie an weiteren psychosozialen und arbeitsbezogenen Kennwerten überprüft werden.

**Methode:**

Die Multicenterstudie an 1010 Personen mit nichtspezifischen CRS (Internationale Klassifikation der Krankheiten und verwandter Gesundheitsprobleme, ICD-10: M51/53/54) untersuchte zu Beginn einer stationären VMO den Unterschied in psychologischen sowie arbeits- und schmerzbezogenen Kennwerten in Abhängigkeit vom Schweregrad (I–IV). Zudem wurden die Häufigkeitsverteilungen der klinisch unauffälligen und auffälligen Fälle in der schmerzspezifischen Selbstwirksamkeit, Depressivität und subjektiven Prognose der Erwerbstätigkeit in Abhängigkeit vom Schweregrad überprüft.

**Ergebnisse:**

Der Schweregradindex trennte die Grade in den psychologischen sowie arbeits- und schmerzbezogenen Kennwerten in erwarteter Richtung. In angeschlossenen paarweisen Vergleichen unterschied sich Grad IV signifikant von den niedrigeren Graden. Personen mit höheren Schweregraden wiesen ungünstige Werte in den psychosozialen Maßen auf und lagen häufiger im auffälligen Bereich.

**Diskussion:**

Die Befunde stützen die Kriteriumsvalidität des Schweregradindex. Das psychosoziale Risikoprofil bei höheren Graden unterstützt sowohl eine frühzeitige schmerzbezogene und psychologische Diagnostik als auch eine gezielte Zuweisung zu bedarfsgerechten interdisziplinären multimodalen Behandlungsangeboten.

**Graphic abstract:**

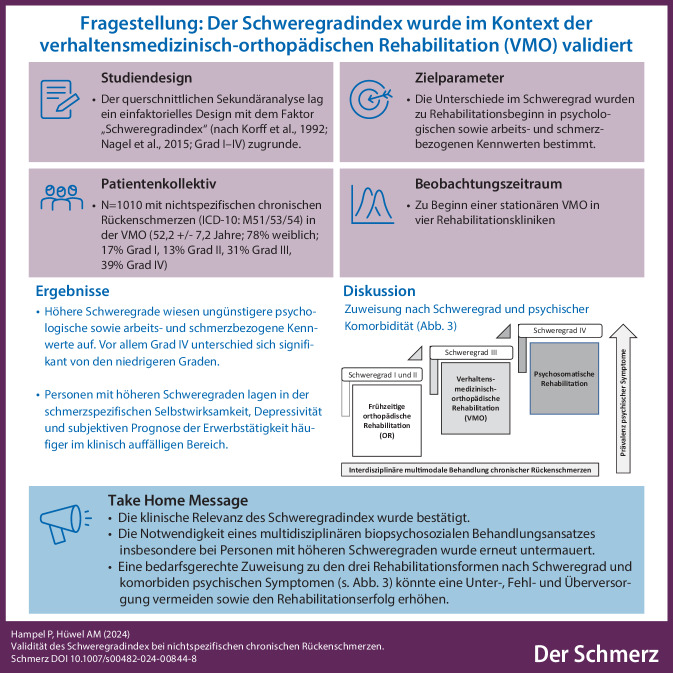

**Zusatzmaterial online:**

Zusätzliche Informationen sind in der Online-Version dieses Artikels (10.1007/s00482-024-00844-8) enthalten.

## Abstract (en)

**Background:**

Higher pain grades are associated with high psychological burden and increase the risk for the persistence of chronic low back pain (CLBP).

**Objectives:**

Previous results on the criterion validity of the Chronic Pain Grade Scale (CPGS) have been extended to the context of inpatient multidisciplinary orthopedic rehabilitation (MOR) and have been supplemented with additional psychosocial and work-related measures.

**Methods:**

In this multicenter study, psychological, work- and pain-related outcomes were examined among 1010 individuals with nonspecific CLBP (ICD-10: M51/53/54) prior to the beginning of an inpatient MOR stratified by pain grade (I–IV). Additionally, frequency distributions of scores regarding pain-specific self-efficacy, depression, and subjective prognosis of gainful employment by pain grade in patients were investigated.

**Results:**

The CPGS differed between all pain grades in the psychological, work- and pain-related outcomes in the expected direction. In post hoc pairwise comparisons, grade IV was significantly different from the lower grades. Patients with higher pain grades showed unfavorable levels in psychosocial parameters and more frequently scores in the clinical range than expected.

**Conclusions:**

These results confirm the criterion validity of the CPGS. The psychosocial risk pattern observed in higher pain grades supports the importance of conducting early pain-related and psychological diagnostic assessments and implementing systematic allocation to needs-based interdisciplinary multimodal treatments.

Höhere Grade chronischer Schmerzen gehen einher mit einer hohen psychischen Belastung. Einer frühzeitigen Diagnostik des Schweregrads und psychischer Beeinträchtigungen kommt daher eine besondere Bedeutung zu. Der Schweregradindex gilt als ein reliables und valides Messinstrument, was jedoch im deutschsprachigen Raum in der rehabilitativen Versorgung chronischer Rückenschmerzen noch nicht validiert wurde.

## Hintergrund

Eine bedeutsame Rolle in der Chronifizierung von Rückenschmerzen spielen psychosoziale Faktoren [14, 15]. So wurde eine erhöhte Auftretenswahrscheinlichkeit von psychischen Beeinträchtigungen wie Depressionen oder Ängsten bei Personen mit chronischen Rückenschmerzen (CRS) festgestellt [2]. Zunehmend findet hierbei die Selbstwirksamkeit mehr Beachtung: Kürzlich wurde sie als zentrales Konzept in der interdisziplinären multimodalen Schmerztherapie (IMST) angeführt [31] und die schmerzspezifische Selbstwirksamkeit erwies sich als vermittelnder Schutzfaktor in der langfristigen Beziehung zwischen Depressivität und arbeitsbezogenen Faktoren bei CRS [11]. Schließlich gelten noch arbeitsbezogene Maße als wichtige Faktoren bei der Schmerzchronifizierung [11]. So war das Risiko für eine Rehabilitation von Rückenschmerzen und eine Erwerbsminderungsrente sowie für die Wahrscheinlichkeit einer dauerhaften Arbeitsunfähigkeit (AU) bei einer ungünstigen subjektiven Prognose der Erwerbstätigkeit (SPE; [24]) erhöht [7].

Nichtspezifische chronische Schmerzen können anhand des Chronifizierungsstadiums und des Schweregrads beschrieben werden. Hierbei bezieht sich die Schweregradeinteilung nach von Korff et al. [20] auf das Ausmaß der Schmerzintensität und der schmerzbedingten Beeinträchtigung. Diese Klassifikation unterscheidet anhand von 7 Items 4 Schweregrade (I = geringe Schmerzintensität, geringe Beeinträchtigung; II = hohe Schmerzintensität, geringe Beeinträchtigung; III = hohe schmerzbedingte Beeinträchtigung, mäßig limitierend; IV = hohe schmerzbedingte Beeinträchtigung, stark limitierend) und hat sich im internationalen Bereich etabliert [17, 21]. Eine Berücksichtigung eines „severity code“ wird auch in der Internationalen Klassifikation der Krankheiten und verwandter Gesundheitsprobleme (ICD-11) empfohlen, jedoch sparsamer operationalisiert [32]. In einer aktuellen prospektiven Studie mit Versichertendaten von Erwerbstätigen mit Rückenschmerzen im Schweregrad III und IV persistierten diese dysfunktionalen Schmerzen bei 48 % der Personen über den 2‑Jahres-Zeitraum [34], sodass Personen mit dysfunktionalen Schmerzen eine besondere Risikogruppe für eine Aufrechterhaltung von CRS darstellen.

Studien in verschiedenen Versorgungssystemen belegten die Konstrukt- und Kriteriumsvalidität des Schweregradindex, indem Maße zur Schmerzintensität und schmerzbedingten Funktionseinschränkung stark assoziiert waren [3, 17, 20, 21, 26]. Zusätzlich zeigte insbesondere der Grad IV erhöhte psychische Beeinträchtigungen wie eine Depressivität und eingeschränkte Lebensqualität [20, 21] sowie häufigere psychosomatische Komorbiditäten [3]. Jedoch stehen Analysen im Rahmen der stationären verhaltensmedizinisch-orthopädischen Rehabilitation (VMO) bislang aus.

## Ziel und Fragestellung

Die vorliegende Sekundäranalyse des Datensatzes des Forschungsprojekts Debora [11, 28] überprüfte die Kriteriumsvalidität der Schweregradklassifikation nach von Korff et al. ([20], modifiziert nach [27]) bei Personen mit nichtspezifischen CRS in der VMO. So wurden hauptsächlich die Unterschiede zu Rehabilitationsbeginn in psychologischen sowie arbeits- und schmerzbezogenen Kennwerten in Abhängigkeit vom Schweregrad untersucht (Hauptfragestellung). Ferner sollten erste Hinweise auf die klinische Relevanz des Schweregradindex anhand der Häufigkeitsverteilungen der klinisch auffälligen Ausprägungen in ausgewählten psychologischen und arbeitsbezogenen Kennwerten in Abhängigkeit vom Schweregrad ermittelt werden (Nebenfragestellung). Hiermit sollten bisherige Validitätsnachweise zum Schweregradindex im Rahmen einer multizentrischen Studie sowohl auf das Setting der VMO als auch auf weitere Variablen wie die schmerzspezifische Selbstwirksamkeit und arbeitsbezogene Kennwerte erweitert werden.

## Methoden

### Stichprobe

Die Stichprobe wurde im Rahmen einer prospektiven Multicenterstudie [11, 28] im Zeitraum von Oktober 2014 bis Dezember 2015 in vier stationären Rehabilitationskliniken mit VMO-Abteilungen rekrutiert. Zu Rehabilitationsbeginn wurden alle Personen mit nichtspezifischen CRS im ärztlichen Aufnahmegespräch über die Studienteilnahme aufgeklärt und gebeten, vor Studienbeginn informierte Einwilligungserklärungen zu unterschreiben. Es wurden Personen im Alter zwischen 20 und 65 Jahren eingeschlossen, die seit mindestens 6 Monaten CRS, eine Hauptdiagnose nach ICD-10 in den Bereichen M51, M53 oder M54 sowie ausreichende deutsche Sprachkenntnisse aufwiesen [11, 19, 28]. Die Studie wurde von der Ethikkommission der Deutschen Gesellschaft für Psychologie genehmigt.

Die Teilnehmenden waren im Mittel 52,2 Jahre alt (SD = 7,2) und zu 78,0 % weiblich (*n* = 788; Tab. 1). Es wurden 303 Personen dem Schweregrad I (*n* = 173; 17,1 %) und II (*n* = 130; 12,9 %) zugeordnet. Die meisten (70 %) befanden sich im Schweregrad III (*n* = 315; 31,2 %) und Schweregrad IV (*n* = 392; 38,8 %).

**Tab. 1:** Stichprobencharakteristik

Kennwerte	Grad I (*n* = 173)	Grad II (*n* = 130)	Grad III (*n* = 315)	Grad IV (*n* = 392)	Gesamt (*N* = 1010)
**Soziodemografische Daten**					
*Alter, Jahre*	53,18	53,60	51,83	51,52	52,17
M ± SD	±6,25	±6,70	±7,08	±7,73	±7,19
*Geschlecht, weiblich*	128	109	244	307	788
*n* (%)	(74,0)	(83,8)	(77,5)	(78,3)	(78,0)
*Verheiratet*^*1*^	102	85	190	224	601
*n *(%)	(60,4)	(66,4)	(61,1)	(58,3)	(60,6)
*Schulabschluss*^*2*^					
*n (%)*					
Haupt‑/Volksschule	22	22	69	90	203
	(12,9)	(17,1)	(22,1)	(23,1)	(20,3)
Realschule	92	61	145	198	496
	(54,1)	(47,3)	(46,5)	(50,8)	(49,6)
(Fach‑)Abitur	52	42	93	93	280
	(30,6)	(32,5)	(29,8)	(23,8)	(27,7)
**Sozialmedizinische Daten**					
*Erwerbstätig*^*3*^	160	124	263	301	848
*n *(%)	(95,8)	(98,4)	(86,5)	(81,4)	(87,7)
*AU-Tage*^*4*^*, ≤2 Wochen*	130	100	164	103	497
*n* (%)	(87,2)	(91,7)	(70,7)	(34,0)	(62,7)
*Work Ability Index*^*5*^					
*Gesamtwert, n *(%)					
Kritisch	31	30	142	275	478
	(3,3)	(3,2)	(15,2)	(29,3)	(51,0)
Mäßig	88	79	134	73	374
	(9,4)	(8,4)	(14,3)	(7,8)	(39,9)
Gut	42	14	20	5	81
	(4,5)	(1,5)	(2,1)	(0,5)	(8,6)
Sehr gut	4	0	0	0	4
	(0,4)	(–)	(–)	(–)	(0,4)
**Schmerzbezogene Daten**					
*Schmerzdauer*^*6*^*, Jahre*	13,78	15,63	14,64	13,63	14,22
M ± SD	±10,33	±11,04	±10,45	±10,36	±10,48
*Anzahl der Schmerzorte*	4,05	5,19	5,25	5,82	5,26
	±1,97	±2,32	± 2,30	±2,73	±2,50
*Chronifizierungsstadium, MPSS*					
*n (%)*					
I	77	42	75	69	263
	(7,6)	(4,2)	(7,4)	(6,8)	(26,0)
II	79	60	174	185	498
	(7,8)	(5,9)	(17,2)	(18,3)	(49,3)
III	17	28	66	138	249
	(1,7)	(2,8)	(6,5)	(13,7)	(24,7)

*M* Mittelwert, *SD* Standardabweichung, *MPSS* Mainzer Stadienmodell der Schmerzchronifizierung (vgl. [19])

^1^*N* = 992

^2^*N* = 1001

^3^*N* = 967

^4^*N* = 793

^5^*N* = 937

^6^*N* = 909

### Messinstrumente

#### Psychologische Kennwerte

Die schmerzbezogene Selbstwirksamkeit wurde mit dem Fragebogen zur Erfassung der schmerzspezifischen Selbstwirksamkeit (FESS) in seiner deutschen Version ermittelt [23]. Die 10 Items wurden auf einer Antwortskalierung von 1 (gar nicht überzeugt) bis 6 (vollkommen überzeugt) eingeschätzt. Die Summenwerte wurden anhand eines Cut-off-Werts von 25,63 dichotomisiert, wobei die Ausgangsstichprobe von Debora (*N* = 1260; *M* = 37,38 ± 11,75) zugrunde gelegt wurde (vgl. [19]). Die Depressivität wurde anhand der Allgemeinen Depressionsskala bestimmt (ADS; [13]); hierbei wurde der Summenwert von 20 Items auf einer Antwortskalierung von 0 (selten oder überhaupt nicht) bis 3 (meistens, die ganze Zeit) berechnet. Werte ab > 22 wurden als klinisch auffällig bewertet. Die beiden Subtests „Physische“ und „Psychische Lebensqualität“ der Short-Form 12 (SF-12) erfassten die gesundheitsbezogene Lebensqualität mit jeweils 6 Items [25].

#### Arbeitsbezogene Kennwerte

Die subjektive Prognose der Erwerbstätigkeit wurde mit der gleichnamigen Skala zur Messung der subjektiven Prognose der Erwerbstätigkeit (SPE) erhoben, die über Einzelitems die derzeitige Berufstätigkeit, Erwerbsfähigkeit und das Rentenbegehren erfragt [24]. Als klinisch auffällig wurden Summenwerte mit >1 eingestuft [7]. Über 2 Einzelitems des Gesamtindex der Arbeitsfähigkeit (Work Ability Index [WAI]) wurden die subjektive physische und psychische Arbeitsfähigkeit abgebildet [12].

#### Schmerzbezogene Kennwerte

Für die Schmerzgraduierung wurden dem Deutschen Schmerzfragebogen (DSF; [27]) 7 Items entnommen und entsprechend zum Schweregradindex verrechnet [20]. Hierbei wurden sie teilweise modifiziert: Zur Bestimmung der Schmerzintensität wurden die momentane, größte und durchschnittliche Schmerzintensität auf einer 11-stufigen Antwortskalierung eingeschätzt. Um Veränderungen unmittelbar nach der Rehabilitation abbilden zu können, betrug der Beobachtungszeitraum zu allen Messzeitpunkten zwei Wochen und nicht wie im DSF vier Wochen. In die Messung der schmerzbedingten Beeinträchtigung bezogen auf die letzten 3 Monate gingen 4 Items ein: Zunächst wurde die Anzahl der Tage erfasst, an denen die Personen schmerzbedingt nicht ihren üblichen Aktivitäten nachgehen konnten. Zudem wurde die schmerzbedingte Beeinträchtigung im Alltag, in der Freizeit und bei der Arbeit auf einer 11-stufigen Antwortskalierung erhoben. Schließlich wurde über die 12 3‑stufigen Items des Funktionsfragebogens Hannover (FFbH‑R; [18]) die subjektiv erlebte Funktionsbeeinträchtigung bei Alltagsaktivitäten durch Rückenschmerzen erhoben. Die erlebte Funktionskapazität erstreckt sich von 0 bis 100 %.

### Auswertung

Die Unterschiede in den psychologischen sowie arbeits- und schmerzbezogenen Kennwerten in Abhängigkeit vom Schweregradindex wurden in dieser Querschnittstudie anhand einfaktorieller multi- bzw. univariater Varianzanalysen mit dem unabhängigen 4fach abgestuften Faktor „Schweregrad“ überprüft (Grad I–IV). Es schlossen sich paarweise Vergleiche (*t*-Tests) mit einer Adjustierung nach Bonferroni an. Da zusätzlich durchgeführte Rangvarianzanalysen die parametrischen Analysen bestätigen konnten, werden hier nur die parametrischen Befunde dargestellt. Um die Häufigkeitsverteilungen der klinisch auffälligen Personen in Abhängigkeit vom Schweregrad zu untersuchen, wurden die ausgewählten Variablen zunächst dichotomisiert und χ^2^-Tests unterzogen.

Insgesamt wurde ein Signifikanzniveau von *p* < 0,05 zugrunde gelegt. Die den Varianzanalysen nachgeschalteten Einzelvergleiche mit kleinen Effektstärken wurden jedoch nicht zur Interpretation herangezogen. Die Effektstärken wurden wie folgt interpretiert (klein, moderat, groß): η^2^: ≥ 0,010, ≥ 0,059, ≥ 0,138; Cohens *d*: ≥ 0,20, ≥ 0,50, ≥ 0,80; Cramers *V* ≥ 0,100, ≥ 0,300, ≥ 0,500 [4].

## Ergebnisse

### Hauptfragestellung

**Abb. 1:**
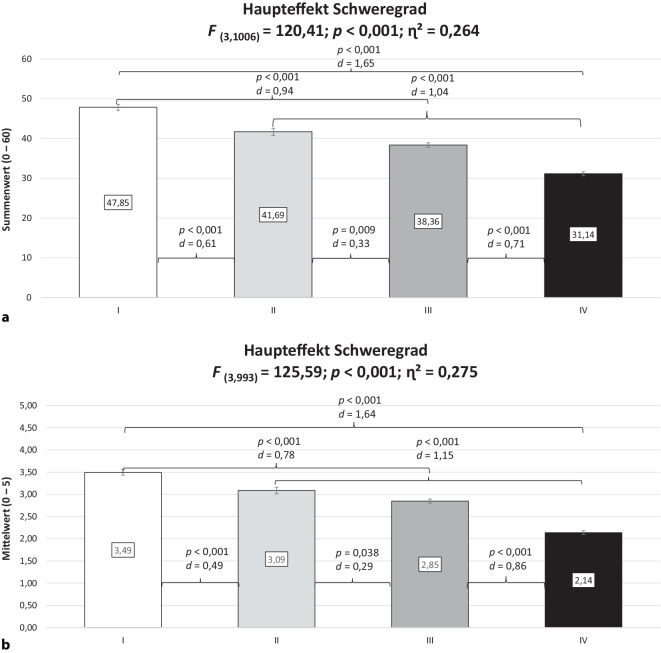
Schmerzspezifische Selbstwirksamkeit (**a**) und physische Arbeitsfähigkeit (**b**) in Abhängigkeit vom Schweregrad (Mittelwert ± Standardfehler*; F* Prüfgröße, *p* statistische Signifikanz, *η*^*2*^ Eta-Quadrat, *d* Cohens *d*, *ns* nicht signifikant [*p* ≥ 0,05]). Schweregrad nach von Korff et al. [20]. (Mod. nach [27])

**Abb. 2:**
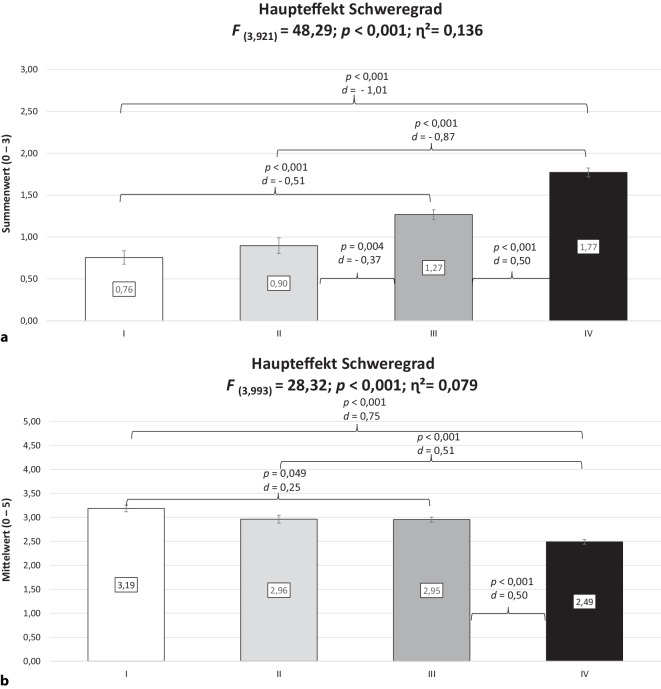
Subjektive Prognose der Erwerbstätigkeit (**a**) und psychische Arbeitsfähigkeit (**b**) in Abhängigkeit vom Schweregrad (Mittelwert ± Standardfehler; *F* Prüfgröße, *p* statistische Signifikanz, *η*^*2*^ Eta-Quadrat, *d* Cohens d, *ns* nicht signifikant [*p* ≥ 0,05]). Schweregrad nach von Korff et al. [20]. (Mod. nach [27])

Die einfaktoriellen Varianzanalysen ergaben signifikante Unterschiede in allen Kennwerten in Abhängigkeit vom Schweregrad, die mit Ausnahme der psychischen Lebensqualität mittlere bis hohe Effektstärken aufwiesen (Online-Zusatzmaterial Tab. S1; Abb. 1 für die schmerzspezifische Selbstwirksamkeit und physische Arbeitsfähigkeit; Abb. 2 für die subjektive Prognose der Erwerbstätigkeit und psychische Arbeitsfähigkeit). Zunächst sollen die Befunde zur durchschnittlichen Schmerzintensität gesondert dargestellt werden, da dieser Kennwert in die Bestimmung des Schweregrads einging. Es zeigten sich für den Grad I verglichen mit allen anderen Graden signifikant geringere Ausprägungen mit großen Effektstärken. Ferner stuften Personen im Grad III ihren durchschnittlichen Schmerz signifikant geringer ein als im Grad IV, was eine moderate Effektstärke aufwies.

Abb. 1 veranschaulicht für die schmerzspezifische Selbstwirksamkeit, dass der Grad IV verglichen mit den Graden I und II signifikant geringere Ausprägungen mit großen Effektstärken aufzeigte. Ferner wiesen Personen im Grad III eine signifikant geringere schmerzspezifische Selbstwirksamkeit mit großer Effektstärke auf als im Grad I. Moderate Effektstärken ergaben sich für die Vergleiche I vs. II bzw. III vs. IV. Für die nicht abgebildete körperliche Lebensqualität konnten diese Befunde bestätigt werden (Online-Zusatzmaterial Tab. S1). Die physische Arbeitsfähigkeit bei Personen im Grad IV war mit großen Effektstärken geringer ausgeprägt als in allen anderen Graden. Eine moderate Effektstärke konnte für den Vergleich I vs. III festgestellt werden.

In Abb. 2 wird ersichtlich, dass Personen im Grad IV verglichen mit den Graden I und II eine signifikant ungünstigere subjektive Prognose der Erwerbstätigkeit mit großen Effektstärken berichteten. Moderate Effektstärken konnten für die Vergleiche I vs. III bzw. III vs. IV aufgezeigt werden. Die psychische Arbeitsfähigkeit war dagegen nur bei Personen im Grad IV signifikant mit moderaten Effektstärken geringer ausgeprägt als in allen anderen Graden.

Ferner zeigte sich für die Funktionskapazität ähnlich zur schmerzspezifischen Selbstwirksamkeit und subjektiven Prognose der Erwerbstätigkeit, dass Personen mit Grad IV verglichen mit den Graden I und II signifikant ungünstigere Ausprägungen mit großen Effektstärken aufwiesen (Online-Zusatzmaterial Tab. S1). Moderate Effektstärken konnten für die Vergleiche I vs. II, I vs. III bzw. III vs. IV festgestellt werden. Die Depressivität und psychische Lebensqualität waren signifikant mit großer bzw. moderater Effektstärke bei Personen im Grad IV ungünstiger ausgeprägt als im Grad I. Moderat wiesen zudem Personen im Grad IV eine signifikant höhere Depressivität auf als im Grad II bzw. III.

### Nebenfragestellung

Insgesamt bestätigte die Überprüfung der Häufigkeitsverteilungen die Befunde zur Hauptfragestellung (Online-Zusatzmaterial Tab. S2). Für die ausgewählten Kennwerte wurden im Grad I mehr unauffällige Fälle als erwartet und im Grad IV mehr auffällige Fälle als erwartet beobachtet. Wie bereits in der Hauptfragestellung differenzierte Grad IV zwischen allen anderen Graden deutlich.

## Diskussion

### Status der Stichprobe

Wie im Setting der VMO zu erwarten war, wies die Mehrheit der vorliegenden Studie höhere Schweregrade auf. Dies stimmt mit deutschen Daten aus schmerztherapeutischen Einrichtungen überein [3, 22], grenzt sich jedoch von Daten der gesetzlichen Krankenversicherung [26], Primärversorgung [17] und Allgemeinbevölkerung ab [30], deren Personen mehrheitlich niedrigere Schweregrade angaben.

### Unterschiede nach Schweregradindex

Insgesamt konnte der Schweregradindex hypothesenkonform zwischen den 4 Graden differenzieren, was besonders für den körperbezogenen Bereich (schmerzspezifische Selbstwirksamkeit, Funktionskapazität, physische Lebensqualität und Arbeitsfähigkeit) belegt werden konnte. Hierbei differenzierte die schmerzspezifische Selbstwirksamkeit sowohl zwischen den Abstufungen der Schmerzintensität (Grad I vs. II) als auch der schmerzbedingten Beeinträchtigung (Grad III vs. IV), was deren Bedeutung nochmals unterstreicht [11, 31]. Da weitere Analysen zeigten, dass die schmerzspezifische Selbstwirksamkeit zwar moderat mit dem Kompetenzerleben (*r* = 0,45, *p* < 0,001), aber klinisch nicht relevant mit der mentalen Ablenkung des Fragebogens zur Erfassung der Schmerzverarbeitung korrelierte (*r* = 0,10, *p* = 0,002, *N* = 1005; [10]), spricht es tatsächlich eher für eine Messung der Kompetenzerwartung [31]. Kennwerte der emotionalen Beeinträchtigung (Depressivität, psychische Arbeitsfähigkeit) waren durch einen moderaten Einfluss des Schweregrads gekennzeichnet, allerdings zeigte sich für die psychische Lebensqualität lediglich eine kleine Effektstärke. Studien zur Validität des Mainzer Stadienmodells der Schmerzchronifizierung in verschiedenen Versorgungssystemen legten auch eine geringere Differenzierungskraft der psychischen Lebensqualität nahe [8, 19]. Gleichfalls übereinstimmend mit den vorliegenden Befunden zu den psychologischen Maßen wurden für die schmerzbezogene Erlebensvermeidung nicht signifikante Einzelvergleiche zwischen den Graden I vs. II und II vs. III bei chronischen Schmerzen berichtet [22]. Schließlich unterstützen die Befunde zum arbeitsbezogenen Kennwert „subjektive Prognose der Erwerbstätigkeit“ die Kriteriumsvalidität der Schmerzgraduierung. Somit erweitert die vorliegende Analyse die bisherigen Ergebnisse zum Einfluss des Schweregradindex auf weitere Schmerzkennwerte [3, 17, 20, 21, 26] sowie die Depressivität und Lebensqualität [20, 21] sowohl auf das Setting der VMO als auch auf die schmerzspezifische Selbstwirksamkeit und arbeitsbezogenen Kennwerte.

### Klinische Relevanz des Schweregradindex

Die vorliegenden Befunde sprechen für die klinische Bedeutsamkeit des Schweregradindex: Die Ergebnisse zur Depressivität und subjektiven Prognose der Erwerbstätigkeit verdeutlichen, dass in beiden Kennwerten von der Gesamtstichprobe die Personen in höheren Schweregraden mit insgesamt 41 bzw. 38 % auffälliger Fälle ein deutlich erhöhtes Risikoprofil zeigten. Dies unterstreicht nochmals übereinstimmend mit früheren Empfehlungen die Notwendigkeit eines multidisziplinären biopsychosozialen Behandlungsansatzes insbesondere bei Personen mit höheren Schweregraden [16].

**Abb. 3:**
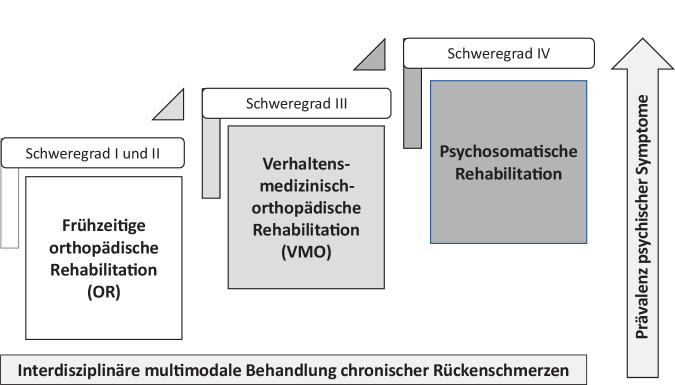
Zuweisungsschema nach Schweregrad und komorbiden psychischen Symptomen

Abschließend ist festzuhalten, dass der etablierte Schweregradindex mit seiner 4fach-Abstufung sehr gut an die Versorgungsstruktur im rehabilitativen Setting angelegt werden kann. Die inhaltlichen Befunde unterstreichen erneut die bedarfsgerechte Zuweisung von Personen mit der Hauptdiagnose nichtspezifischer CRS zu den unterschiedlichen Rehabilitationsformen, um eine Unter‑, Fehl- und Überversorgung zu vermeiden [14] sowie die Effektivität der Maßnahmen zu erhöhen (Abb. 3; vgl. [28]): Personen mit geringer schmerzbedingter Beeinträchtigung sollten der orthopädischen Rehabilitation zugewiesen werden, jedoch Personen mit hoher schmerzbedingter Beeinträchtigung bei mäßiger Einschränkung der VMO und bei starker Einschränkung der psychosomatischen Rehabilitation. Unter einer biopsychosozialen Perspektive findet hierdurch auch Berücksichtigung, dass psychische Komorbiditäten mit der Zunahme des Schweregrads assoziiert sind. So steht in der VMO die Primär- und Sekundärprävention psychischer Symptome im Vordergrund und es erfolgt eine psychotherapeutische Mitbehandlung der psychischen Symptome bei CRS [5, 33]. Umfangreichere psychologische Interventionen wie schmerzpsychotherapeutische Maßnahmen kommen jedoch erst in der psychosomatischen Rehabilitation zum Einsatz und haben die Tertiärprävention psychischer Symptome zum Ziel (vgl. [6]). Hierdurch werden auch Empfehlungen der ICD-11 [32] und zur Gesundheitsversorgung chronischer Schmerzen [14] angemessen adressiert.

In neuerer Zeit wurde die Bedeutung der psychosomatischen Sichtweise bei chronischen Schmerzen noch mehr in den Vordergrund gerückt. Hierbei wurde jedoch eher die Behandlung somatoformer Störungen und hier die nach ICD-10 verschlüsselte anhaltende Schmerzstörung (F45.4-) einbezogen. So stellten Bach und Simhandl [1] zur ganzheitlichen Behandlung chronischer Schmerzen ein psychosomatisches Prozessmodell mit 3 Stufen vor, das neben einer Basisversorgung und Schmerzbewältigung noch eine spezielle Schmerzpsychotherapie bei ausgeprägter Affektregulationsstörung bzw. Körperbeziehungsstörung umfasst, die noch durch eine Biographiearbeit ergänzt wird. Frey und Schiltenwolf [9] thematisieren die Notwendigkeit, neben den etablierten psychologischen Maßnahmen zur Förderung der Selbstwirksamkeit und Veränderung dysfunktionaler Schmerzkognitionen noch die Bindungsstile mit zu berücksichtigen. Zudem wird betont, dass die IMST bei schweren Fällen von chronischen Schmerzen erwogen werden sollte, wenn sich bislang durch die unimodalen Maßnahmen keine entsprechenden Behandlungserfolge eingestellt haben. Die IMST, die im deutschen Operationen- und Prozedurenschlüssel (OPS) insbesondere unter 8‑918 klassifiziert wird, erfolgt im deutschsprachigen Bereich zumeist im stationären und wenig im teilstationären Bereich [9, 29]. Um darüber hinaus nicht nur Personen mit einer bereits fortgeschrittenen Chronifizierung zu versorgen, wurden erste Ansätze für den ambulanten Bereich vorgestellt und untersucht, die auch die Bedürfnisse von Personen mit einem erhöhten Chronifizierungsrisiko adressieren (vgl. auch [15]). Somit kann zukünftig eine verbesserte Umsetzung multidisziplinärer biopsychosozialer Behandlungsansätze in der Versorgung chronischer Schmerzen erwartet werden.

### Limitationen

Durch die Verwendung von Filtervariablen reduzierte sich die Stichprobe um 296 Personen. Drop-out-Analysen zeigten jedoch, dass sich die verbliebenen von den ausgeschlossenen Personen nur in den AU-Zeiten, mit einer kleinen Effektstärke, unterschieden. Ferner ist der Querschnittsansatz zu kritisieren, aber Längsschnittstudien konnten auch die prognostische Validität des Schweregradindex belegen [3, 20]. Die vorliegenden Befunde wurden zwar in einer multizentrischen Studie mit einer repräsentativen Stichprobe ermittelt, jedoch sind sie auf Personen in einer stationären VMO mit erhöhter Chronifizierung und höheren Schweregraden beschränkt.

## Fazit für die Praxis

- Die klinische Relevanz des Schweregradindex wurde bestätigt. Ca. 40 % von der Gesamtstichprobe wiesen höhere Schweregrade und klinisch auffällige Ausprägungen in der Depressivität und subjektiven Prognose der Erwerbstätigkeit auf. Dies unterstreicht die Notwendigkeit eines multidisziplinären biopsychosozialen Behandlungsansatzes insbesondere bei Personen mit höheren Schweregraden.
- Der Schweregradindex ist ein kurzes, valides Messinstrument, das zur Therapiesteuerung eingesetzt werden kann.
- Eine bedarfsgerechte Zuweisung zu den drei Rehabilitationsformen in Abhängigkeit von Schweregrad und komorbiden psychischen Symptomen ist erneut indiziert, um eine Unter‑, Fehl- und Überversorgung zu vermeiden sowie den Rehabilitationserfolg zu erhöhen.

## Supplementary Information

Tab. S1: Deskriptive Statistik und F‑Statistik für die Kennwerte in Abhängigkeit vom Schweregrad

Tab. S2: Häufigkeitsausprägungen in den Kennwerten in Abhängigkeit vom Schweregrad

## Data Availability

Daten werden auf begründete Anfrage zur Verfügung gestellt. Data will be made available on reasonable request.
