# Supplementary material for: Validity of the Chronic Pain Grade Scale in nonspecific chronic low back pain
Source: Schmerz. 2024 Nov 7;40(1):46–55. [Article in German] doi: 10.1007/s00482-024-00844-8 (PMC12858461; doi:10.1007/s00482-024-00844-8)
Supplement: Supplementary file 1 — Tab. S1: Deskriptive Statistik und F‑Statistik für die Kennwerte in Abhängigkeit vom Schweregrad [file 482_2024_844_MOESM1_ESM.pdf]

Online-Zusatzmaterial Tab. S1 Deskriptive Statistik und F-Statistik für die Kennwerte in Abhängigkeit vom Schweregrad nach von Korff et al. (1992; modifiziert nach Nagel et al., 2015) für *N* = 1010

		**Schweregrad**				**F-Statistik**				**Paarweise Vergleiche**						
		**Grad I (*n*=173)**	**Grad II (*n*=130)**	**Grad III (*n*=315)**	**Grad IV (*n*=392)**						**I vs. II**	**I vs. III**	**I vs. IV**	**II vs. III**	**II vs. IV**	**III vs. IV**
Schmerzspezifische Selbstwirksamkeit (FESS)	***M***	47,85	41,69	38,36	31,14	***df***	3,1006	***p***	< ,001	***p***	< ,001	< ,001	< ,001	,009	< ,001	< ,001
	SE	0,77	0,89	0,57	0,51	***F***	120,41	**η²**	,264	***d***	0,61	0,94	1,65	0,33	1,04	0,71
Depressivität (ADS)	***M***	17,74	21,16	22,09	28,40	***df***	3,1006	***p***	< ,001	***p***	,028	< ,001	< ,001	*ns*	< ,001	< ,001
	SE	0,79	0,91	0,59	0,53	***F***	50,91	**η²**	,132	***d***	-0,33	-0,42	-1,02	-0,09	-0,70	-0,61
Körperliche Lebensqualität (SF-12)[1]	***M***	45,23	40,05	37,04	31,24	***df***	3,1006	***p***	< ,001	***p***	< ,001	< ,001	< ,001	< ,001	< ,001	< ,001
	SE	0,57	0,66	0,43	0,38	***F***	151.23	**η²**	,311	***d***	0,69	1,08	1,85	0,40	1,17	0,77
Psychische Lebensqualität (SF-12)[1]	***M***	42,30	39,70	38,76	35,09	***df***	3,1006	***p***	< ,001	***p***	*ns*	,003	< ,001	*ns*	< ,001	< ,001
	SE	0,81	0,93	0,60	0,54	***F***	20,75	**η²**	,058	***d***	0,24	0,33	0,68	0,09	0,43	0,34
Subjektive Prognose der Erwerbstätigkeit (SPE)	***M***	0,76	0,90	1,27	1,77	***df***	3,921	***p***	< ,001	***p***	*ns*	< ,001	< ,001	,004	< ,001	< ,001
	SE	0,08	0,09	0,06	0,05	***F***	48,29	**η²**	,136	***d***	-0,14	-0,51	-1,01	-0,37	-0,87	-0,50
Physische Arbeitsfähigkeit (WAI)[2]	***M***	3,49	3,09	2,85	2,14	***df***	3,993	***p***	< ,001	***p***	< ,001	< ,001	< ,001	,038	< ,001	< ,001
	SE	0,06	0,07	0,05	0,04	***F***	49,44	**η²**	,275	***d***	0,49	0,78	1,64	0,29	1,15	0,86
Psychische Arbeitsfähigkeit (WAI)[2]	***M***	3,19	2,96	2,95	2,49	***df***	3,993	***p***	< ,001	***p***	*ns*	,049	< ,001	*ns*	< ,001	< ,001
	SE	0,07	0,08	0,05	0,05	***F***	28,32	**η²**	,079	***d***	0,24	0,25	0,75	0,01	0,51	0,50
Funktionskapazität (FFbH-R)	***M***	79,96	70,83	67,00	53,39	***df***	3,1006	***p***	< ,001	***p***	< ,001	< ,001	< ,001	*ns*	< ,001	< ,001
	SE	1,31	1,51	1,00	0,90	***F***	109,24	**η²**	,246	***d***	0,53	0,75	1,54	0,22	1,01	0,79
Durchschnittliche Schmerzintensität (DSF)	***M***	2,86	5,58	4,97	5,96	***df***	3,1006	***p***	< ,001	***p***	< ,001	< ,001	< ,001	< ,001	*ns*	< ,001
	SE	0,12	0,14	0,09	0,08	***F***	157,19	**η²**	,319	***d***	-1,71	-1,32	-1,96	0,38	-0,24	-0,62

M Mittelwert, ***SE*** Standardfehler, ***df*** Freiheitsgrade, ***F*** Prüfgröße, ***p*** statistische Signifikanz, **η^2** Eta-Quadrat, ***d*** Cohens *d*, **ns** nicht signifikant ($p \geq ,05$), **FESS** Fragebogen zur Erfassung der schmerzspezifischen Selbstwirksamkeit, **ADS** Allgemeine Depressionsskala, **SF-12** Short Form-12, **SPE** Subjektive Prognose der Erwerbstätigkeit, **WAI** Work-Ability-Index, **FFbH-R** Funktionsfragebogen Hannover, **DSF** Deutscher Schmerzfragebogen; [1]MANOVA$_{SF\text{-}12}$ $F_{(6,2010)} = 89,74$, $p < ,001$, $\eta^2 = ,211$), [2]MANOVA$_{WAI}$ $F_{(6,1984)} = 60,33$, $p < ,001$, $\eta^2 = ,154$).
