# Supplementary material for: Validity of the Chronic Pain Grade Scale in nonspecific chronic low back pain
Source: Schmerz. 2024 Nov 7;40(1):46–55. [Article in German] doi: 10.1007/s00482-024-00844-8 (PMC12858461; doi:10.1007/s00482-024-00844-8)
Supplement: Supplementary file 2 — Tab. S2: Häufigkeitsausprägungen in den Kennwerten in Abhängigkeit vom Schweregrad [file 482_2024_844_MOESM2_ESM.docx]

Online-Zusatzmaterial Tab. S2 Häufigkeitsausprägungen in den Kennwerten in Abhängigkeit vom Schweregrad nach von Korff et al. (1992; modifiziert nach Nagel et al., 2015) für *N*= 1010

|  | | **Schweregrad** | | | | |
| --- | --- | --- | --- | --- | --- | --- |
|  |  | **Grad I (*n*=173)** | **Grad II (*n*=130)** | **Grad III (*n*=315)** | **Grad IV (*n*=392)** | **∑**  **(*N* = 1010)** |
| FESS - **Schmerzspezifische Selbstwirksamkeit** (χ² _(3, N = 1010)_ = 112,13, *p* < ,001, *V* = ,333) | | | | | | |
| Unauffällig  (RW ≥ 25.63) | Anzahl (%) | 169 (16,7) | 124 (12,3) | 276 (27,3) | 263 (26,3) | 832 (82,4) |
|  | Erwartete Anzahl | 143 | 107 | 260 | 323 | 832 |
| Auffällig  (RW < 25.63) | Anzahl (%) | 4 (0,4) | 6 (0,6) | 39 (3,9) | 129 (12.8) | 178 (17,6) |
|  | Erwartete Anzahl | 31 | 23 | 56 | 69 | 178 |
| ADS - **Depressivität** (χ² _(3, N = 1010)_ = 96,17, *p* < ,001, *V* = ,309) | | | | | | |
| Unauffällig  (RW ≤ 22) | Anzahl (%) | 119 (11,8) | 75 (7,4) | 174 (17,2) | 115 (11.4) | 483 (47,8) |
|  | Erwartete Anzahl | 83 | 62 | 151 | 188 | 483 |
| Auffällig  (RW > 22) | Anzahl (%) | 54 (5,3) | 55 (5,4) | 141 (14,0) | 277 (27,4) | 527 (52,2) |
|  | Erwartete Anzahl | 90 | 68 | 164 | 205 | 527 |
| SPE- **Subjektive Prognose der Erwerbstätigkeit** (χ² _(3, N = 925)_ =100,81, *p* < .001, *V* = ,330) | | | | | | |
| Unauffällig  (RW ≤ 1,00) | Anzahl (%) | 118 (12,8) | 83 (9,0) | 166 (17,9) | 122 (13,2) | 489 (52,9) |
|  | Erwartete Anzahl | 83 | 61 | 156 | 189 | 489 |
| Auffällig  (RW > 1,00) | Anzahl (%) | 38 (4,1) | 33 (3,6) | 129 (13,9) | 236 (25,5) | 436 (47,1) |
|  | Erwartete Anzahl | 74 | 55 | 139 | 169 | 436 |

**RW**Rohwert, **χ²** Chi-Quadrat, ***p*** statistische Signifikanz, ***V*** Cramers *V*.
